# *Leishmania* infection and blood food sources of phlebotomines in an area of Brazil endemic for visceral and tegumentary leishmaniasis

**DOI:** 10.1371/journal.pone.0179052

**Published:** 2017-08-24

**Authors:** Antônia Suely Guimarães-e-Silva, Soraia de Oliveira Silva, Rosa Cristina Ribeiro da Silva, Valéria Cristina Soares Pinheiro, José Manuel Macário Rebêlo, Maria Norma Melo

**Affiliations:** 1 Centro de Estudos Superiores de Caxias, Laboratório de Entomologia Médica (LABEM), Universidade Estadual do Maranhão, Caxias, Maranhão, Brazil; 2 Departamento de Parasitologia, Laboratório de Biologia de *Leishmania*, Instituto de Ciências Biológicas, Universidade Federal de Minas Gerais, Belo Horizonte, Minas Gerais, Brazil; 3 Departamento de Biologia, Laboratório de Entomologia e Vetores, Universidade Federal do Maranhão, São Luís, Maranhão, Brazil; National Centre For Cell Science, INDIA

## Abstract

The aims of the study were to determine the blood feeding preferences of sandflies and to identify species of *Leishmania* that infected phlebotomines in Caxias, Maranhão, Brazil, an area that is highly endemic for leishmaniasis. Sandflies were captured in light traps located in the peridomiciliary environments of randomly selected houses in urban and rural settings between 1800 and 0600 hours on new moon days between March 2013 and February 2015. DNA extracts from 982 engorged female sandflies were submitted to fragment length polymorphism analysis to identify infecting species of *Leishmania*, and blood sources were identified for 778 of these specimens. Infection by *Leishmania infantum* was detected in *Lutzomyia longipalpis*, *Lu*. *whitmani* and *Lu*. *termitophila; L*. *infantum/L*. *braziliensis* in *Lu*. *longipalpis*, *Lu*. *whitmani* and *Lu*. *trinidadensis; L*. *shawi* in *Lu*. *longipalpis; L*. *mexicana* in *Lu*. *longipalpis; L*. *braziliensis* in *Lu*. *longipalpis* and *Lu*. *whitmani*; *L*. *guyanensis* in *Lu*. *longipalpis* and *Lu*. *termitophila*; *L*. *amazonensis* in *Lu*. *longipalpis* and *L*. *lainsoni* or *L*. *naiffi* in *Lu*. *longipalpis*, while *Lu*. *longipalpis* and *Lu*. *trinidadensis* were infected with unidentified *Leishmania* sp. Blood sources were identified in 573 individual phlebotomines and the preferred hosts were, in decreasing order, chicken, dog, rodent and human with lower preferences for pig, horse, opossum and cattle. *Lu*. *longipalpis* and *Lu*. *whitmani* performed mixed feeding on man, dog and rodent, while *Lu*. *longipalpis* was the most opportunistic species, feeding on the blood of all hosts surveyed, but preferably on dog/chicken, dog/rodent and rodent/chicken. Our findings reveal the concomitant circulation of *Leishmania* species that cause visceral leishmaniasis and tegumentary leishmaniasis in the study area, and explain the occurrence of autochthonous human cases of both clinical forms of leishmaniasis in Caxias, Maranhão. The results support our hypothesis that, in the municipality of Caxias, transmission of *Leishmania* occurs in close proximity to humans.

## Introduction

The leishmaniases are a group of neglected tropical diseases caused by protozoa of the genus *Leishmania* (Kinetoplastida: Trypanosomatidae) and transmitted by hematophagous sandflies of the subfamily Phlebotominae (Diptera: Psychodidae)[1]. The diseases are endemic to countries in Europe, Middle East, Southeast Asia and Americas, where they are considered a public health problem [2]. The epidemiology of leishmaniasis depends on the species of the parasite, the ecological characteristics of the transmission area and the presence of phlebotomine vectors and their reservoirs, which can include wild, synanthropic and domestic animals [3]. In the Americas, leishmaniasis occurs as two main clinical types, namely American visceral leishmaniasis (AVL), the causative agent of which is *Leishmania (Leishmania) infantum*, and American tegumentary leishmaniasis (ATL), which manifests in cutaneous, mucocutaneous and diffuse forms [3,4].

In Brazil, the principal vectors of the AVL pathogen are *Lutzomyia longipalpis*, an anthropophilic phlebotomine that is widely distributed throughout the country, and *Lu*. *cruzi*, which is prevalent in some municipalities in the central-west region [5,6]. The major reservoirs of *L*. *infantum* are domestic dogs and other wild canids [3].

Regarding ATL, Brazil is the country with the greatest variety *Leishmania* species responsible for tegumentary forms of the disease in humans. Six of these species belong to the subgenus *Viannia*: *L*. (*Viannia*) *braziliensis*, *L*. (*V*.) *guyanensis*, *L*.(*V*.) *lainsoni*, *L*.(*V*.) *naiffi*, *L*.(*V*.) *shawi* and *L*.(*V*.) *lindenbergi*, while *L*.(*Leishmania*) *amazonensis* belongs to the subgenus *Leishmania* [3,7]. The species of sandflies associated with transmission of ATL are *Lu*. *intermedia*, *Lu*. *neivai*, *Lu*. *whitmani*, *Lu*. *umbratilis*, *Lu*. *flaviscutellata*, *Lu*. *antunesi*, *Lu*. *migonei*, *Lu*. *fischeri*, *Lu*. *pessoai*, *Lu*. *wellcomei*, *Lu*. *complexa*, *Lu*. *ayrozai*, *Lu*. *paraensis*, *Lu*. *amazonensis*, *Lu*. *hirsuta hirsuta*, *Lu*. *ubiquitalis*, *Lu*. *gomezi* and *Lu*. *tuberculata* [8].

Although the distribution of *Leishmania* species within Brazil is not yet fully know, the geographical spread of leishmaniasis has expanded over recent years in several states, including the northeastern state of Maranhão where AVL and ATL are endemic and widespread [9]. Phlebotomine sandflies naturally infected with species of *Leishmania* have already been identified in Maranhão as, for example, *Lu*. *whitmani* infected with *Leishmania* sp. [10] and *Lu*. *longipalpis* infected with *L*. *infantum* [11,12]. According to Nascimento et al. [12], the frequency of *Lu*. *longipalpis* naturally infected with the AVL pathogen was 0.76% in the municipality of Caxias, Maranhão, where both AVL and ATL are prevalent. Despite this low infection rate, AVL endemicity in the area was sustained as demonstrated by the notification of 45 human cases of the disease during the year of the study [13]. In addition, 10 cases of ATL were detected during the same period, but the species of *Leishmania* responsible were not identified.

Considering that 91 phlebotomine species are distributed within the different ecosystems of Maranhão [9], it is very important to identify the *Leishmania* sp. (especially those that cause leishmaniasis in man) associated with these sandflies and to determine the blood food sources of the infected insects. Molecular techniques afford high sensitivity in detecting *Leishmania* sp. and identifying the blood source of infected phlebotomines. Such information facilitates the understanding of vector competence, transmission dynamics and the ecoepidemiology of leishmaniasis, thereby increasing our knowledge of how species interact with their natural habitat and assisting in the guidance of disease control and surveillance [14–17].

Few studies have focused on the identification of species of *Leishmania* that infect phlebotomines and on the blood food sources of these vectors in the cerrado area of Caxias, Maranhão. Our results support the hypothesis that, in the municipality of Caxias, the transmission of *Leishmania* occurs in environs close to human dwellings. We have verified this hypothesis by carrying out an ecological study in which we have determined the frequency of phlebotomine species and identified the host preferences of the sandflies in urban and rural settings of Caxias.

## Materials and methods

### Ethics statement

Details of the project were submitted to and approved by the Ethics Committee of the Universidade Estadual do Maranhão (protocol no. 909.095). The collection of blood samples from the marsupial (*Didelphis albiventris*) was authorized by the Ethics Committee on the Use of Animals in Research of the Universidade Federal de Minas Gerais (license no. 37950–1), while the collection of phlebotomines was approved by the Instituto Chico Mendes de Conservação da Biodiversidade (authorization no. 46319–1).

### Study area

**Fig 1:**
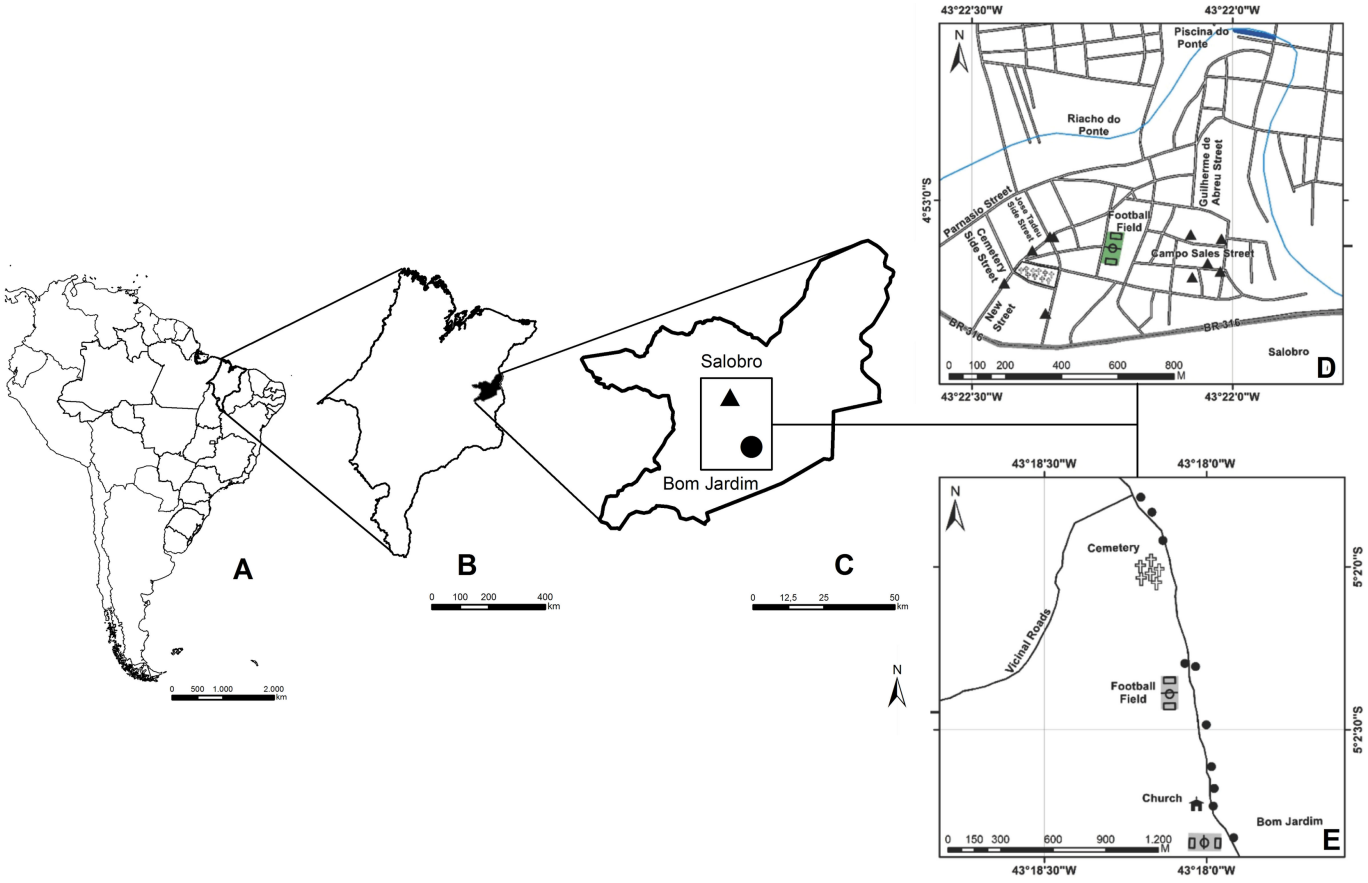
Locations of sampling sites in Caxias, Maranhão, Brazil. **A)** Location of the Brazil country in South America, **B**) Location of Maranhão State (MA) in Brazil; **C**) Location of the municipality of Caxias in MA; **D**) Sampling sites in Salobro (▲) and **E**) Sampling sites in Bom Jardim (●). (Source: **IBGE System Coordinate UTM—FUSE 23 Datum SAD 2017).**

The study was conducted in Caxias (04°51'22 "S, 43°21'22" W; altitude 66 m) located in the eastern mesoregion of Maranhão State. The municipality comprises an area of 5,150,647 km^2^ with a population of 159,326 inhabitants [18]. The warm semi-humid and semi-arid climate corresponds to categories Aw and As, respectively, in the Köppen classification (Fig 1). The area is characterized by a distinct rainy season from January to June (mean temperature 26.1°C) and a dry season between July and December (mean temperature 35.6°C) [19], and the vegetation is typical of a savanna region.

### Sampling and identification of phlebotomines

The entomological study was carried out on new moon days during the period March 2013 to February 2015 with collections performed from 1800 to 0600 hours. Phlebotomines were captured in the vicinity of 20 houses selected at random in the urban neighborhood of Salobro (*n* = 10) and in the rural district of Bom Jardim (*n* = 10). Sampling was performed near the shelters of domestic animals, when present, with the aid of Centers for Disease Control (CDC)-type light traps placed at a height of 1.5m above ground.

Captured sandflies were killed by freezing and screened. Female specimens, engorged or not, were dissected, mounted onto slides with Berlese fluid and examined under an optical microscope (400 X magnification). All phlebotomines were identified according to the Young and Duncan [20] classification system. Samples of thorax and abdomen from dissected sandflies were stored in a freezer at -20°C until required for DNA extraction.

### DNA extraction

DNA was extracted using the Gentra Puregene Blood Kits (Qiagen, Valencia, CA, USA), used according to the manufacturer's instructions, and extracts were stored at -20°C until required for analysis by polymerase chain reaction (PCR). In order to serve as a control for the extraction procedure, and to exclude the possibility of PCR inhibition, DNA samples were submitted to PCR analysis using the primers 5'-GTGGCCGAACATAATGTTAG-3' and 5'-CCACGAACAAGTTCAACATC-3' to amplify a 200 base pair (bp) fragment from the cacophony gene IVS6 region in New World sandflies [21]. A positive control from laboratory-reared *Lu*. *longipalpis*, and a negative control comprising a reaction mixture without DNA, were used in the amplifications.

### Identification of *Leishmania* species

DNA samples was screened for the presence of *Leishmania* DNA by amplification of a 300–350 bp fragment of the internal transcribed spacer 1 (*ITS1*) using the primers 5’CTGGATCATTTTCCGATG3’ (LITSR) and 5’TGATACCACTTATCGCACTT’3 (L5.8S) [22,23]. PCR products were submitted to electrophoresis on 5% polyacrylamide gel [24], and the gels were visualized by staining with silver nitrate [25]. The ITS1-PCR amplicons were submitted to restriction fragment length polymorphism (RFLP) analysis (*Hae*III digestion) and the band patterns were compared with those obtained from the DNA of the World Health Organization *Leishmania* strains reference *L*. *amazonensis* (IFLA/BR/1967/PH8), *L*. *guyanensis* (MHOM/BR/1975/M1176), *L*. *braziliensis* (MHOM/BR/1975/M2903), *L*. *lainsoni* (MHOM/BR/81/M6426), *L*. *naiffi* (MDAS/BR/1979/M5533), *L*. *shawi* (MCEB/BR/1984/M84408), *L*. *mexicana* (HMOM/BZ/1982/BEL21) and *L*. *infantum* (MHOM/1973/BH46), which had been characterized in the Laboratory of *Leishmania* Biology, Instituto de Ciências Biológicas, Universidade Federal de Minas Gerais.

### Identification of phlebotomine blood food sources

In order to test for the presence of DNA from the blood of vertebrate hosts, samples of sandfly DNA were submitted to PCR analysis using PuRe Taq Ready-To-Go^™^ PCR beads (Amersham Biosciences, São Paulo, Brazil) and primers described previously [26] for the amplification of a 359 bp fragment of the mitochondrial cytochrome b (*cytB*) gene. The *cytB*-PCR amplicons were submitted to RFLP analysis (*Hae*III and *Mbo*I digestion). Amplification and restriction products were subjected to electrophoresis on 5% polyacrylamide gel [24], and the gels were visualized by staining with silver nitrate [25]. The band patterns were compared with those obtained from the DNA of vertebrate blood. Positive controls consisted of DNA extracted from blood of opossum (*Didelphis albiventris*) (sample made available by the Universidade Federal de Minas Gerais), pig (*Sus domesticus*), cattle (*Bos taurus*), human (*Homo sapiens*), rodent (*Mus musculus*), dog (*Canis familiaris*), chicken (*Gallus domesticus*) and horse (*Equus caballus*) (samples made available by the Universidade Federal do Maranhão).

### *Leishmania* and phlebotomine blood food sources analysis

To assure reproducible results, all reactions were performed at least in three independent experiments. The gels were carefully analyzed to determine the PCR-RFLP restriction patterns. To perform data analysis the LabImage-1D Gel Analysis software, Version 2.7.2 (Copyright 1999–2004, Kapelan GmbH, Halle, Saale, Germany), available at the website www.labimage.net, was employed. This software is able to determine the size of each DNA fragment (band) selected in the gel constitutive of each DNA band in comparison with the fragments of the 25 bp and 50 bp DNA ladder (Invitrogen, USA).

## Results

The 200 bp-fragment from the cacophony IVS6 region of phlebotomines could be amplified in all of the sandfly DNA samples studied, thus confirming the high quality of the extracted DNA and the absence of PCR inhibitors.

Of the 982 female phlebotomines dissected, 769 were collected in urban settings and comprised the species *Lu*. *longipalpis* (*n* = 763), *Lu*. *evandroi* (*n* = 3), *Lu*. *sordellii* (*n* = 1), *Lu*. *lenti* (*n* = 1) and *Lu*. *whitmani* (*n* = 1), while 213 were collected in the rural area and comprised the species *Lu*. *longipalpis* (*n* = 175), *Lu*. *whitmani* (*n* = 23), *Lu*. *trinidadensis* (*n* = 6), *Lu*. *termitophila* (*n* = 5), *Lu*. *lenti* (*n* = 3) and *Lu*. *sordellii* (*n* = 1).

**Fig 2:**
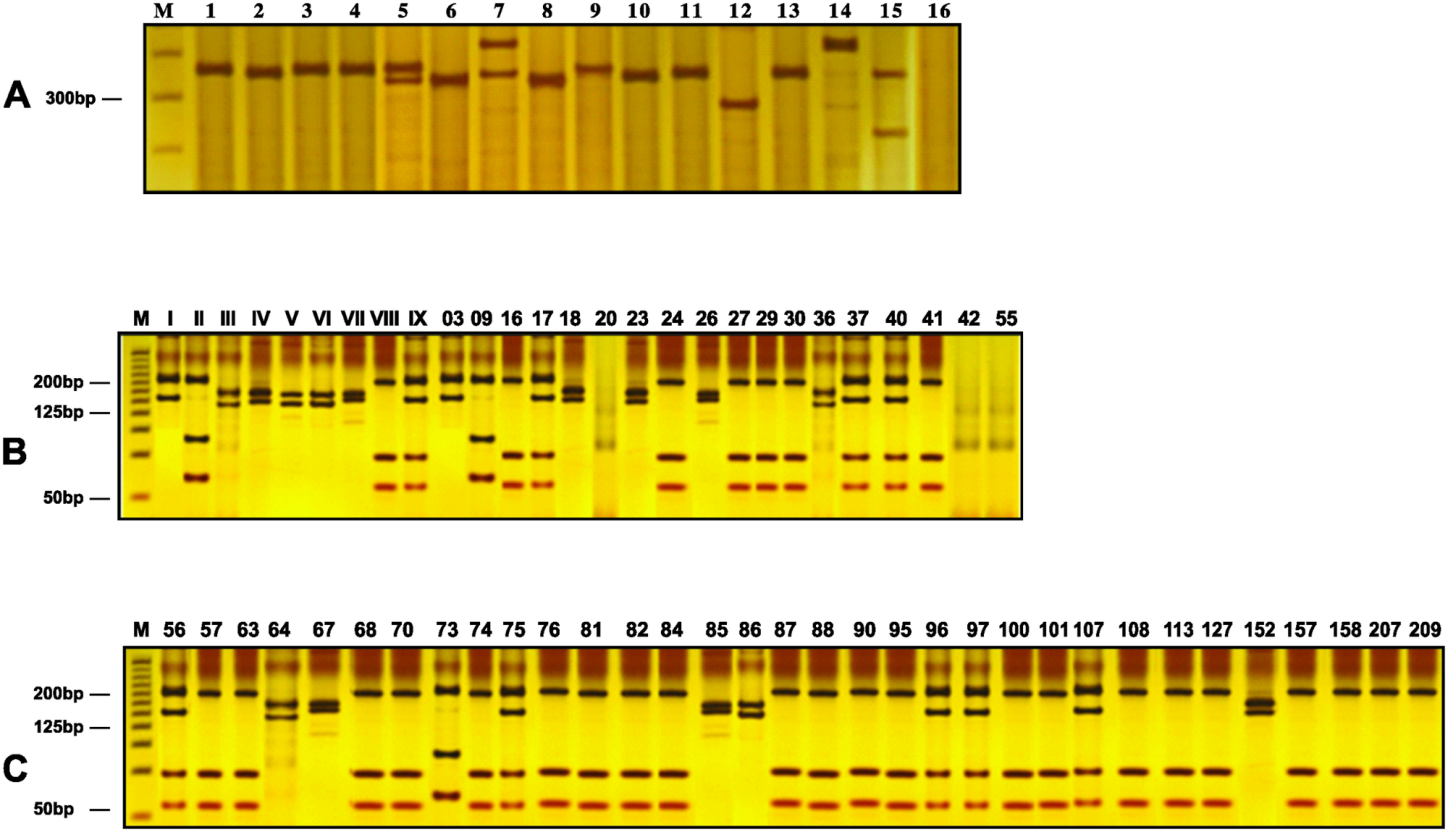
Internal transcribed spacer 1 (*ITS1*) gene amplicons from *Leishmania* DNA and restriction fragment length polymorphism (RFLP) analyses. Silver-stained 5% polyacrylamide gels showing: **A**) ITS1-PCR: Lane M—Promega standard molecular weight ladder (100 bp), Lane 1—positive control—amplicon from DNA of *Leishmania infantum* promastigotes, Lanes 2–15—amplicons from DNA sandfly samples collected in Caxias, Maranhão, Brazil, Lane 16—negative control; **B**) ITS1-PCR-RFLP (*Hae*III digestion): Lane M—Invitrogen standard molecular weight ladder (25 bp), Lanes I-IX—DNA from positive controls, I—*L*. *amazonensis*, II—*L*. *mexicana*, III–*L*. *guyanensis*, IV- *L*. *braziliensis*, V–*L*. *lansoni*, VI–*L*. *naiffi*, VII—*L*. *shawi*, VIII—*L*. *infantum*, IX—*L*. *infantum/L*. *braziliensis*, Lanes 3–55—DNA from phlebotomine samples **C**) ITS1-PCR-RFLP (*Hae* III digestion): Lane M: Invitrogen standard molecular weight ladder (25 bp), Lanes 56–209—DNA from phlebotomine samples.

According to ITS1-PCR analysis, 51 of the 982 female sandflies were positive for *Leishmania* sp. presenting an overall infection rate of 5.2% (Table 1, Fig 2A). The species of *Leishmania* identified by ITS1-PCR-RFLP were *L*. *infantum* (*n* = 28), mixed *L*. *infantum/L*. *braziliensis* (*n* = 8), *L*. *braziliensis* (*n* = 3), *L*. *shawi* (*n* = 3), *Leishmania* sp. (*n* = 3), *L*. *mexicana* (*n* = 2), *L*. *guyanensis* (*n* = 2), *L*. *amazonensis* (*n* = 1) and *L*. *lainsoni* or *L*. *naiffi* (*n* = 1) (Table 1, Fig 2B and 2C, S1 Table). In the urban area, the infection rate was 3.1% (24/769) and was limited to *Lu*. *longipalpis*, with infection by *L*. *infantum* being the most frequent. In the rural area, the overall infection rate was 12.7% (27/213), but rates varied substantially among species of sandfly with *Lu*. *termitophila* presenting the highest frequency of infection (40.0%), followed by *Lu*. *trinidadensis* (33.0%). *Lu*. *whitmani* (17.0%) and *Lu*. *longipalpis* (11.0%). Infection by *L*. *infantum* and mixed infection by *L*. *infantum/L*. *braziliensis* were the most frequent.

**Table 1:** Frequency of *Leishmania*-positive sandflies in the municipality of Caxias, Maranhão, Brazil (March 2013–February 2015) as determined by ITS1-RFLP.

*Lutzomyia* species			*Leishmania* species								
	Sampled females	*Leishmania* positive females	*L*. *infantum*	*L*. *braziliensis*	*L*. *infantum/L*. *braziliensis*	*L*. *shawi*	*L*. *mexicana*	*L*. *guyanensis*	*L*. *amazonensis*	*L*. *lansoni* or *L*. *naiffi*	*Leishmania* sp.
	N	N (%)	N (%)	N (%)	N (%)	N(%)	N (%)	N (%)	N (%)	N (%)	N (%)
**Urban area**											
*Lu*. *longipalpis*	763	24 (3.1)	13 (1.7)	0	5 (0.7)	1 (0.1)	1 (0.1)	0	1 (0.1)	1 (0.1)	2 (0.3)
*Lu*. *evandroi*	3	0	0	0	0	0	0	0	0	0	0
*Lu*. *sordellii*	1	0	0	0	0	0	0	0	0	0	0
*Lu*. *lenti*	1	0	0	0	0	0	0	0	0	0	0
*Lu*. *whitmani*	1	0	0	0	0	0	0	0	0	0	0
Total	769	24 (3.1)	13 (1.7)	0	5 (0.7)	1 (0.1)	1 (0.1)	0	1 (0.1)	1 (0.1)	2 (0.3)
**Rural area**											
*Lu*. *longipalpis*	175	19 (11.0)	12 (7.0)	1 (0.6)	1 (0.6)	2 (1.0)	1 (0.6)	1 (0.6)	0	0	0
*Lu*. *whitmani*	23	4 (17.0)	2 (9.0)	1 (4.0)	1 (4.0)	0	0	0	0	0	0
*Lu*. *trinidadensis*	6	2 (33.0)	0	0	1 (16.7)	0	0	0	0	0	1 (16.7)
*Lu*. *termitophila*	5	2 (40.0)	1 (20.0)	0	0	0	0	1 (20.0)	0	0	0
*Lu*. *lenti*	3	0	0	0	0	0	0	0	0	0	0
*Lu*. *sordellii*	1	0	0	0	0	0	0	0	0	0	0
Total	213	27 (12.7)	15 (7.0)	2 (0.9)	3 (1.4)	2 (1.1)	1 (0.5)	2 (0.9)	0	0	1 (0.5)
**Overall Frequency**	**982**	**51 (5.2)**	**28 (2.9)**	**2 (0.2)**	**8 (0.8)**	**3 (0.3)**	**2 (0.2)**	**2 (0.2)**	**1 (0.1)**	**1 (0.1)**	**3 (0.3)**

**Fig 3:**
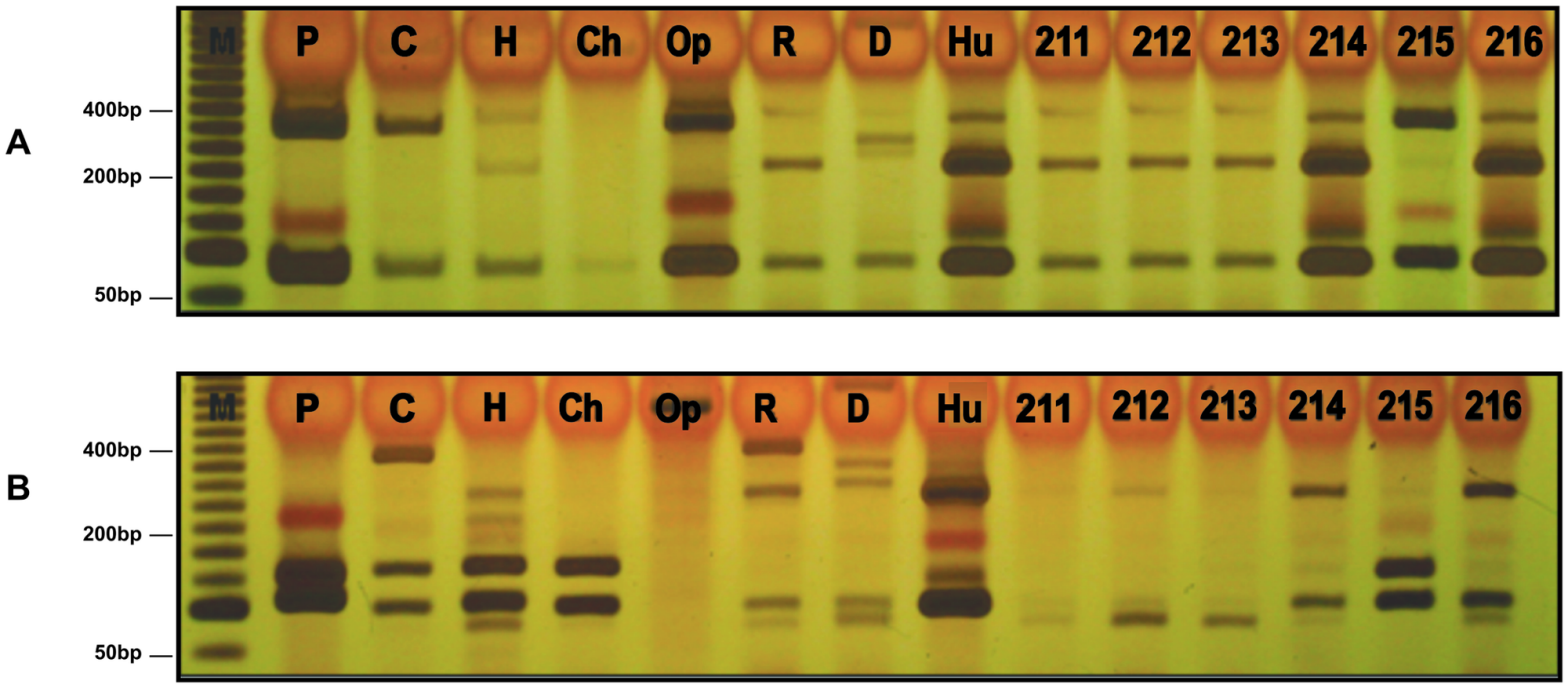
Restriction fragment length polymorphism (RFLP) analyses of mitochondrial cytochrome b (*cytB*) gene amplicons from *Leishmania* DNA. Silver-stained 5% polyacrylamide gels showing: **A**) CytB-PCR-RFLP (*Mbo*I digestion) and **B**) CytB-PCR-RFLP (*Mbo*I digestion). Lanes M: Invitrogen standard molecular weight ladder (50 bp), Lanes P-H DNA from positive controls, P—pig, C—cattle, H—horse, Ch—chicken, Op—opossum, R—rodent, D—dog, Hu—man; Lanes 211–216—DNA from *Leishmania*-positive phlebotomine samples from urban and rural areas.

Sources of blood feed could be identified in 73.7% (573/778) of engorged female sandflies, most of which were collected in the urban area (63.4%; 363/573). The *cytB*-PCR-RFLP band patterns following *Mbo*I and *Hae*III digestion (Fig 3A and 3B, respectively) showed that 475 sandflies had fed on a single host, while 98 had fed on a combination of two different hosts (mixed feeding).

Considering sandflies that had simple feeding (Table 2), food source preferences in the urban area were chicken (44.9%), dog (28.9%), rodent (10.6%) and human (9.0%), whereas in the rural area the preferential hosts were dog (46.6%), chicken (30.5%), rodent (9.8%) and human (8.6%). Taking the two areas together, the decreasing order of preference was: chicken (39.6%), dog (35.4%), rodent (10.3%), human (8.8%), pig (2.5%), horse (2.3%), opossum (0.8%) and cattle (0.2%).

**Table 2:** Frequency of engorged sandflies found to have fed on one vertebrate blood source (simple feeding) in the municipality of Caxias, Maranhão, Brazil (March 2013—February 2015) as determined by mitochondrial *cytB* PCR-RFLP.

*Lutzomyia* species	Vertebrate host								
	Dog	Human	Rodent	Chicken	Cattle	Pig	Horse	Opossum	Total
	N (%)	N (%)	N (%)	N (%)	N (%)	N (%)	N (%)	N (%)	N
**Urban area**									
*Lu*. *longipalpis*	85 (97.7)	26 (96.3)	31 (96.9)	135 (100)	0	9 (100)	9 (100)	2 (100)	297
*Lu*. *evandroi*	2 (2.3)	1 (3.7)	0	0	0	0	0	0	3
*Lu*. *lenti*	0	0	1 (3.13)	0	0	0	0	0	1
Total	87 (28.9)	27 (9.0)	32 (10.6)	135 (44.9)	0	9 (3.0)	9 (3.0)	2 (0.7)	301
**Rural area**									
*Lu*. *longipalpis*	66 (81.5)	12 (80.0)	16 (94.1)	49 (92.5)	1 (100)	3 (100)	2 (100)	2 (100)	151
*Lu*. *whitmani*	8 (9.9)	2 (13.3)	1 (5.9)	4 (7.5)	0	0	0	0	15
*Lu*. *termitophila*	3 (3.7)	0	0	0	0	0	0	0	3
*Lu*. *trinidadensis*	3 (3.7)	1 (6.7)	0	0	0	0	0	0	4
*Lu*. *sordellii*	1 (1.2)	0	0	0	0	0	0	0	1
Total	81 (46.6)	15 (8.6)	17 (9.8)	53 (30.5)	1 (0.6)	3 (1.7)	2 (1.1)	2 (1.1)	174
**Overall frequency**	**168 (35.4)**	**42 (8.8)**	**49 (10.3)**	**188 (39.6)**	**1 (0.2)**	**12 (2.5)**	**11 (2.3)**	**4 (0.8)**	**475**

With regard to sandflies that had fed on two hosts (mixed feeding) (Table 3), preferences for food source combinations in the urban area were dog/chicken (48.5%), dog/rodent (27.3%), rodent/chicken (10.6%), human/chicken (6.1%) and human/rodent (3.0%), whereas in the rural area the preferential host combinations were dog/rodent (43.8%), dog/chicken (37.5%), rodent/chicken (9.4%), human/rodent (6.3%) and human/chicken (3.1%). Considering the two areas together, the decreasing order of combined food source preference was: dog/chicken (44.9%), dog/rodent (32.7%), rodent/chicken (10.2%), human/chicken (5.1%), human/rodent (4.1%), and rodent/horse, rodent/pig and human/opossum (1.0% each).

**Table 3:** Frequency of engorged sandflies found to have fed on two vertebrate blood sources (mixed feeding) in the municipality of Caxias, Maranhão, Brazil (March 2013—February 2015) as determined by mitochondrial *cytB* PCR-RFLP.

*Lutzomyia* species	Vertebrate host combinations								
	Dog/rodent	Human/rodent	Human/Chicken	Rodent/chicken	Dog/chicken	Rodent/horse	Rodent/pig	Human/opossum	Total
	N (%)	N (%)	N (%)	N (%)	N (%)	N (%)	N (%)	N (%)	N
**Urban area**									
*Lu*. *longipalpis*	17 (94.4)	2 (100)	4 (100)	7 (100)	32 (100)	1 (100)	1 (100)	1 (100)	65
*Lu*. *whitmani*	1 (5.6)	0	0	0	0	0	0	0	1
Total	18 (27.3)	2 (3.0)	4 (6.1)	7 (10.6)	32 (48.5)	1 (1.5)	1 (1.5)	1 (1.5)	66
**Rural area**									
*Lu*. *longipalpis*	6 (42.9)	2 (100)	1 (100)	3 (100)	9 (75,0)	0	0	0	21
*Lu*. *whitmani*	4 (28.6)	0	0	0	3 (25,0)	0	0	0	7
*Lu*. *trinidadensis*	2 (14.3)	0	0	0	0	0	0	0	2
*Lu*. *termitophila*	1 (7.1)	0	0	0	0	0	0	0	1
*Lu*. *lenti*	1 (7.1)	0	0	0	0	0	0	0	1
Total	14 (43.8)	2 (6.3)	1 (3.1)	3 (9.4)	12 (37.5)	0	0	0	32
**Overall frequency**	**32 (32.7)**	**4 (4.1)**	**5 (5.1)**	**10 (10.2)**	**44 (44.9)**	**1 (1.0)**	**1 (1.0)**	**1 (1.0)**	**98**

Females of *Lu*. *longipalpis* fed on the blood of all of the vertebrate hosts studied but showed a single source preference for, in decreasing order, chicken, dog, rodent and human. Furthermore, this phlebotomine fed on eight host combinations in the urban area and five host combinations in the rural area, with the predominance of dog/chicken followed by dog/rodent. In the rural area, *Lu*. *whitmani* exhibited a single source preference for human, dog, chicken, and rodent blood, in decreasing order, and for dog/rodent and dog/chicken combinations. The only example of an engorged *Lu*. *whitmani* female captured in the urban area has fed on a dog/rodent combination. The single source preferences of *Lu*. *evandroi* and *Lu*. *lenti* in the urban area were dog and rodent, respectively, while dog was the preferential host for *Lu*. *termitophila*, *Lu*. *trinidadensis* and *Lu*. *sordellii* in the rural area. A dog/rodent combination was established as the blood source for *Lu*. *trinidadensis*, *Lu*. *termitophila* and *Lu*. *lenti* that had fed on two hosts in the rural area. As shown in Table 4, the principal sources of *Leishmania*-infected blood in engorged sandflies were dog, rodent and human, independent of the number of vertebrate hosts or *Leishmania* species.

**Table 4:** Frequencies of *Leishmania*-infected sandflies and their respective vertebrate blood sources in the municipality of Caxias, Maranhão, Brazil (March 2013—February 2015).

*Lutzomyia species*	*Leishmania* species and blood sources								
	*L*. *infantum*	*L*. *braziliensis*	*L*. *infantum/* *L*. *braziliensis*	*L*. *shawi*	*L*. *mexicana*	*L*. *guyanensis*	*L*. *amazonensis*	*L*. *lainsoni* or *naiffi*	*Leishmania* sp.
	N (%)	N (%)	N (%)	N (%)	N (%)	N (%)	N (%)	N (%)	N (%)
**Urban area**									
*Lu*. *longipalpis*	5(35.7); dog	1(100); dog	1(20); dog	1(100); dog	1(100); human/chicken		1(100); rodent	1(100); dog	1(100); rodent
	1(7.1); rodent		2(20); dog/rodent						1(50); rodent/chicken
	1(7.1); chicken		1(20); rodent/chicken						
	2(14.2); human								
	1(7.1); dog/rodent								
	2(14.3); rodent/chicken								
	1(7.1); rodent/pig								
**Total**	**14**	**1**	**4**	**1**	**1**	**0**	**1**	**1**	**2**
**Rural area**									
*Lu*. *longipalpis*	4(25.0); dog	1(50); human	1(50); dog		1(50); dog	1(50); chicken			
	14(25.0); chicken				1(50); rodent				
	1(6.3); human								
	2(12.5); dog/rodent								
	1(6.3); chicken/human								
*Lu*. *whitmani*	1(6.3); dog/rodent	1(50); dog							
	1(6.3); dog/chicken								
*Lu*. *termitophila*	1(6.3); dog					1(50); others			
*Lu*. *trinidadensis*			1(50); human						
**Total**	**16**	**2**	**2**	**0**	**2**	**2**	**0**	**0**	**0**

## Discussion

Between March 2013 and February 2015, 107 human and 2,380 canine cases of AVL were recorded in the municipality of Caxias, together with 62 human cases of ATL [13]. The high prevalence of human leishmaniasis may be explained by the numerous cases of *Leishmania* infection in the canine reservoirs coupled with the *L*. *infantum-*infection rates in *Lu*. *longipalpis* (AVL vector) of 2.0 and 7.0% in urban and rural areas, respectively, and the *L*. *braziliensis-*infection rate in *Lu*. *whitmani* (ATL vector) of 4.0% [11,27,28].

In the area encompassed by the present study, *L*. *infantum* infection was detected in the AVL vector *Lu*. *longipalpis* and in non-vectorial species such as *Lu*. *whitmani* and *Lu*. *termitophila*. The natural infection of *Lu*. *longipalpis* by *L*. *infantum* has been described in various AVL-endemic regions of Brazil [29–33], including São Luís, the capital of Maranhão State [11], and the provincial town of Caxias [12]. Indeed, a number of researchers have noted that the transmission of AVL in Maranhão is chiefly associated with the presence of *Lu*. *longipalpis* and of infected canine reservoirs, which are widely distributed in the State [33–35].

In nature, the infection rate of *Lu*. *longipalpis* by *L*. *infantum* is low, even in areas with high AVL endemicity. However, based on the high degree of anthropophilia, vectorial capacity, simultaneous presence of vector and disease, and the abundance and spatial distribution of the vector observed in the present study, it is possible to state with certainty that *Lu*. *longipalpis* plays a key role in the AVL transmission cycle in the municipality of Caxias where prevalence rates of human and canine cases are elevated.

Infection of *Lu*. *whitmani* and *Lu*. *termitophila* by *L*. *infantum* was detected for the first time in the rural area of Caxias during the present study. This finding is important since *Lu*. *whitmani* was the second most abundant species of sandfly in the study area, particularly in the rural setting. On the other hand, the high rate of infection (20%) of *Lu*. *termitophila* by *L*. *infantum* must be examined carefully since only one specimen was infected. Natural infection of *Lu*. *whitmani* and *Lu*. *termitophila* by *L*. *infantum* was first recorded by Saraiva et al. [29] in the state of Minas Gerais. There are also reports of *L*. *infantum* naturally infecting the phlebotomines *Lu*. *migonei* and *Lu*. *cortelezzii*, both species of which are vectors of ATL in Brazil while the latter is a proven vector of ATL in Argentina [36–38].

The finding of infection by *L*. *infantum* in sandflies that are not considered to be vectors of AVL demonstrates the ability of a range of phlebotomine species to acquire *L*. *infantum* infection. The importance of these parasite-phlebotomine associations, even if they occur only occasionally, requires further study in order fully to understand their implications regarding the ecoepidemiology of leishmaniasis. However, the conclusion that a species of sandfly is a vector in the leishmaniasis cycle cannot be based exclusively on the occurrence of natural infection by the causative agent. Together with the natural infection factor, a vector of *Leishmania* sp. must fit the criteria described by Killick-Kendrick [39].

We report here for the first time the occurrence of *Lu longipalpis* naturally infected by *L*. *braziliensis*, *L*. *amazonensis*, *L*. *mexicana*, *L*. *shawi*, *L*. *guyanensis* and *L*. *lainsoni* or *L*. *naiffi* in Maranhão, in an area of cerrado that is not within the Amazon region. However, it is important to emphasize that *Lu longipalpis* was the most abundant and widespread sandfly species in the study area and appeared to be perfectly adapted to domestic and anthropic settings.

The presence of *Lu longipalpis* naturally infected by *L*. *braziliensis* has been described in other regions of Brazil [32,40], while the occurrence of *Lu*. *longipalpis* naturally infected by *L*. *amazonensis* has been established in the state of Mato Grosso do Sul [41,42]. *L*. *amazonensis* is the causative agent of ATL, including the anergic or diffuse cutaneous forms of the disease, and its vectors are normally *Lu*. *flaviscutellata* and *Lu*. *olmeca* in the Brazilian Amazon region [43]. These two phlebotomines were not detected in Caxias during the study period, although human cases of diffuse cutaneous leishmaniasis attributed to *L*. *amazonensis* have been reported in all geographical and ecological regions of Maranhão [44,45].

While the occurrence of *Lu*. *longipalpis* infected by *L*. *mexicana* has not been previously reported in Brazil, *L*. *mexicana-*infected humans have occasionally been recorded [46]. There are no records of *Lu*. *longipalpis* naturally infected by *L*. *shawi*, neither in Maranhão nor in other Brazilian regions, suggesting that this phlebotomine does not form part of the *L*. *shawi* transmission cycle outside the Amazon region, where the normal vector is *Lu*. *whitmani*. Although we found *Lu*. *longipalpis* infected by *L*. *guyanensis* in the rural area of Caxias, this species of *Leishmania* is usually restricted to recently colonized areas of the Amazon region, where *Lu*. *umbratilis* and *Lu*. *anduzei* are the main vectors [46] and edentulous and marsupial mammals are the reservoirs [47,48]. The presence of *L*. *guyanensis* has also been detected in Minas Gerais in a pool of sandflies that are not considered to be vectors of ATL [31].

The presence of *L*. *lainsoni*, or possibly *L*. *naiffi*, is reported here for the first time outside the Amazon region of Brazil. The main vector of *L*. *lainsoni* in the Amazon is *Lu*. *ubiquitalis*, although there are reports of the occurrence of *L*. *lainsoni*-infected *Lu*. *davisi* in the state of Amazonas [49–52] and of *L*. *lainsoni*-infected *Lu*. *nuneztovari anglesi* in Bolivia [53]. Moreover, cases of human infection by this parasite have been recorded in the subandean zone of Peruvian [54]. These findings demonstrate the widespread distribution of *L*. *lainsoni* in South America, which has now been shown to extend to Caxias in the state of Maranhão. The main vectors of *L*. *naiffi*, a *Leishmania* generally found in the Amazon region, are *Lu*. *squamiventris*, *Lu*. *paraensis* and *Lu*. *ayrozai* [55]. Although this parasite has also been found to infect *Lu*. *davisi* and *Lu*. *hirsuta hirsuta*, none of these sandflies have been identified in the Caxias area [56,57].

The presence of *L*. *braziliensis*-infected *Lu*. *whitmani* was confirmed at the site of the present study. This phlebotomine is a natural vector of *L*. *braziliensis* in various Brazilian regions [46], especially in the northeast [27,28], and has been reported previously in various municipalities in Maranhão, including Caxias [58], generally in association with ATL infections. In fact, *L*. *braziliensis* is distributed throughout the ATL-endemic zone of Brazil, from north to south of the country and in both new and well-established settlements, and is commonly associated with the presence of a range of domestic, synanthropic and wild animals.

The presence of *Lu*. *longipalpis*, *Lu*. *whitmani* and *Lu*. *trinidadensis* co-infected by *L*. *infantum*/*L*. *braziliensis*, examples of which have not been recorded recently, is reported herein for the first time in Caxias. There is, however, no evidence that *Lu*. *whitmani* and *Lu*. *trinidadensis* are involved in the transmission of *L*. *infantum* or that *Lu*. *trinidadensis* plays a role in the transmission of *L*. *braziliensis* to humans. Although reports of mixed infections are available, including that of *Lu*. *flaviscutellata* by *L*. *braziliensis* and *L*. *amazonensis* [59], we believe that the occurrence and frequency of natural infection by different species of *Leishmania* are more common than the literature would suggest, particularly in foci where the two species overlap.

In the present study, three sandfly females were classified as infected by *Leishmania* sp., which can be attributed to the presence of unidentified species of *Leishmania* or other trypanosomatids. These problems could not be resolved using the PCR-RFLP technique owing to the small amount of DNA available or to the presence of degraded DNA

The blood food sources of female sandflies were identified in the present study since this information can improve our knowledge regarding *Leishmania* transmission, interactions between *Leishmania* vectors and reservoirs and the anthropophilic attraction of vectors [60,61]. Furthermore, such knowledge could help to clarify the factors associated with the protective or detrimental role of some animals regarding the risk of AVL or ATL transmission to humans in endemic areas [62]. For example, the presence of cattle in *L*. *donovani* transmission areas has been associated with increased risk to humans in some studies and with decreased risk in others, showing that the relationship between mammals and phlebotomines is complex and can affect the abundance, aggregation, feeding behavior and infection rates of the vector [63].

In the present study, *Lu*. *longipalpis* was the most opportunistic species, since it fed on the blood of all vertebrate hosts tested, but exhibited a preference for chickens, dogs rodents and humans, and, to a lesser extent, pigs and horses. Chickens were also the preferential source of blood for female sandflies when all identified species were considered together. Chickens are very common in rural and urban settings, and chicken coops represent attractive areas for phlebotomines to rest and reproduce [64–66]. Although chickens do not act as reservoirs for *Leishmania*, they may be important in the maintenance and domiciliation of vectors by attracting mammalian reservoirs to the vicinity [65]. It is possible that the main role of chickens in the epidemiology of leishmaniasis is to attract sandflies to the peridomicile [14]. Galati et al. [67] called attention to the risk imposed by the presence of chicken coops and pigsties in the peridomicile because they promote breeding and favor the maintenance of high density phlebotomine populations. For this reason, such features should be taken into account by the authorities when planning the surveillance and control of leishmaniasis. Interestingly, the infection of a pig by a species of *Leishmania* has already been detected in Maranhão [68].

Although horses were not high on the preference list of phlebotomine food sources, the intense movement of these animals between rural villages, and even in the urban periphery, could be a risk factor for leishmaniasis. Indeed, clinical cases of naturally acquired leishmaniasis have occasionally been found in horses in transmission areas in Brazil, specifically São Paulo [69] and Minas Gerais [70], and in Venezuela [71].

Considering mixed blood feeding, the dog/rodent combination was the most frequent blood source for female sandflies. This type of feeding behavior reflects the adaptability of sandflies and indicates that these insects can adjust their feeding patterns according to availability, accessibility, abundance, size and biomass of vertebrate hosts [72]. Under certain circumstances, especially in peridomestic and domestic settings, domesticated and synanthropic animals can act as sources of infection for *Leishmania* vectors [73].

Preferences for dogs, rodents and humans, along with the observed mixed feeding behavior, constitute an important epidemiological factor in Caxias since, in our study, *Lu*. *longipalpis* and *Lu*. *whitmani* were found to be infected by *L*. *infantum* and *L*. *braziliensis*, and *Lu longipalpis* and *Lu*. *trinidadensis* were co-infected by *L*. *infantum/L*. *braziliensis*. We also observed that, occasionally, *Lu*. *evandroi* and *Lu*. *sordellii* performed single feeding on dogs and *Lu*. *lenti* fed on rodents, either alone or in combination with dogs, but without infection by *Leishmania*. From an epidemiological viewpoint, it would appear that these species of phlebotomine are not involved in the *Leishmania* transmission cycle in Caxias.

Among domestic animals, dogs are the species most commonly affected by leishmaniasis and have long been recognized as the main domestic reservoirs of *L*. *infantum*. Since infection rates are commonly high among canine populations in endemic areas, and a large proportion of affected animals are asymptomatic [74], dogs represent the top target for AVL control in Brazil [75]. Furthermore, dogs and rodents are considered likely reservoirs of *L*. *braziliensis* in Brazil [76–78], even though the role and epidemiological significance of dogs in this context have yet to be fully understood. Although the presence of *L*. *braziliensis* in dogs has been amply described [76–79], and dogs infected by *L*. *amazonensis* [80] or co-infected by *L*. *infantum/L*. *braziliensis* [81] have been reported in southeast Brazil, the rates of infection by species of *Leishmania* that cause ATL are always lower in dogs than in humans. It may be that dogs, similar to humans, are victims of the disease and not just carriers of parasites in nature.

Results of a study conducted in northeastern Brazil strongly indicated that some small rodents are involved in the maintenance of *L*. *braziliensis* in nature [82], reinforcing the need to study these animals as possible reservoirs of *Leishmania* sp. since they represent an important link between domestic and wild environments [83].

Our observation that *Lu longipalpi*s fed on the blood of opossum in the peridomicile, even at low frequency, is most interesting because it establishes a connection between the domestic and wild cycles of AVL and, according to Cabrera et al. [84], may give rise to an increase in the risk of canine infection by up to 2.6 times.

In conclusion, the present study has demonstrated that *Lu*. *longipalpis* and *Lu*. *whitmani* feed on human blood as well as on the blood of one or more hosts (i.e. dog, rodent, chicken and pig) that inhabit the environs of rural and urban dwellings. Such findings provide strong evidence that these phlebotomines exhibit anthropophilic behavior and support our original hypothesis that, in the municipality of Caxias, transmission of *Leishmania* occurs in close proximity to humans (i.e. inside or near domiciles). However, the observations reported herein of natural infection of non-vectorial sandflies by *Leishmania* sp. do not prove the participation of such species in the transmission of AVL and ATL in Caxias.

In Brazil, the transmission cycles of leishmaniasis have been found to have a focal distribution in specific geographic areas. Leishmaniasis is characterized by complex and specific epidemiological profiles for each local of transmission, involving several species of *Leishmania*, with the potential to cause different clinical manifestations, which can be transmitted by different vectors and maintained by several mammal reservoirs. In spite of the high number of human cases and considering that current control and prevention methods have not been shown to be effective, new strategies for appropriate intervention and control should be developed to avoid propagating even to non-endemic areas. From this perspective, has become increasingly important to study essential issues involved in the transmission, such as the natural sand fly infection rates of the vectors by *Leishamania* sp., the geographical distribution of these parasites in potential vectors and the study of their blood meal sources, which are important tools for eco-epidemiological studies of leishmaniasis.

Our findings have important implications relating to the epidemiology of leishmaniasis especially regarding vector competence, knowledge of which is vital for successful vector surveillance and control.

## Supporting information

S1 Table *Leishmania* species identification based on ITS1 PCR products digestion with restriction enzyme *HaeIII*. (XLSX)
